# Nipah Virus Contamination of Hospital Surfaces during Outbreaks, Bangladesh, 2013–2014

**DOI:** 10.3201/eid2401.161758

**Published:** 2018-01

**Authors:** Md Zakiul Hassan, Hossain M.S. Sazzad, Stephen P. Luby, Katharine Sturm-Ramirez, Mejbah Uddin Bhuiyan, Mohammed Ziaur Rahman, Md Muzahidul Islam, Ute Ströher, Sharmin Sultana, Mohammad Abdullah Heel Kafi, Peter Daszak, Mahmudur Rahman, Emily S. Gurley

**Affiliations:** icddr,b, Dhaka, Bangladesh (M.Z. Hassan, H.M.S. Sazzad, K. Sturm-Ramirez, M.U. Bhuiyan, M.Z. Rahman, M.M. Islam, M.A.H. Kafi, E.S. Gurley);; Stanford University, Stanford, California, USA (S.P. Luby);; Centers for Disease Control and Prevention, Atlanta, Georgia, USA (K. Sturm-Ramirez, U. Ströher);; Institute of Epidemiology, Disease Control and Research, Dhaka (S. Sultana, M. Rahman);; EcoHealth Alliance, New York, New York, USA (P. Daszak)

**Keywords:** Hospital surface contamination, hospital-acquired infection, Nipah virus, viral RNA shedding, infection control, viruses, Bangladesh, bats, paramyxoviruses, United States, encephalitis

## Abstract

Nipah virus (NiV) has been transmitted from patient to caregivers in Bangladesh presumably through oral secretions. We aimed to detect whether NiV-infected patients contaminate hospital surfaces with the virus. During December 2013–April 2014, we collected 1 swab sample from 5 surfaces near NiV-infected patients and tested surface and oral swab samples by real-time reverse transcription PCR for NiV RNA. We identified 16 Nipah patients; 12 cases were laboratory-confirmed and 4 probable. Of the 12 laboratory-confirmed cases, 10 showed NiV RNA in oral swab specimens. We obtained surface swab samples for 6 Nipah patients; 5 had evidence of NiV RNA on >1 surface: 4 patients contaminated towels, 3 bed sheets, and 1 the bed rail. Patients with NiV RNA in oral swab samples were significantly more likely than other Nipah patients to die. To reduce the risk for fomite transmission of NiV, infection control should target hospital surfaces.

Nipah virus (NiV) is a batborne paramyxovirus (*1*,*2*) that causes encephalitis in humans. NiV has caused outbreaks almost every year in Bangladesh since 2001; the case-fatality rate is >70% (*3*).The 2 primary pathways of NiV transmission in Bangladesh are drinking raw date palm sap contaminated with excretions from *Pteropus* spp. fruit bats and human-to-human transmission through close contact with infected persons (*4*–*7*). Nearly one third of identified Nipah patients in Bangladesh were infected through person-to-person transmission (*8*); most of these were family caregivers who provided hands-on care to Nipah patients at home and in hospital (*3*,*6*,*9*,*10*).

Transmission of NiV in hospital settings was first identified in 2001 during an outbreak in Siliguri, India, and in several outbreaks in Bangladesh since 2004 (*6*,*9*,*11*–*13*). In the outbreak in Siliguri, 66 persons were infected, and of the 60 for whom exposure was known, 45 (75%) acquired infection during their hospital stay (11 patients admitted for other illness, 25 hospital staff, and 8 persons who visited an infected patient) (*11*). In Bangladesh, during the 2010–2011 Nipah outbreak, 2 hospital staff (1 physician, 1 hospital cleaner) were infected (*12*,*13*).

NiV RNA has repeatedly been identified in infected patients’ oral secretions (*14*,*15*), and epidemiologic evidence suggests that exposure to respiratory secretions is a likely route of NiV transmission from patient to caregiver (*6*). In 2004, during an NiV outbreak with person-to-person transmission in Bangladesh, NiV RNA was found on a hospital wall near where an NiV patient received care (*6*).

Hospital wards in Bangladesh are often overcrowded with patients, family caregivers, and visitors and have a median of 4 persons/10 m^2^ of floor space (*16*). The floor is often soiled with bodily secretions, and a median of 5 uncovered coughs or sneezes per 10 m^2^ per hour has been observed (*16*). Most wards have intermittent water supply, lack functioning handwashing stations, and have an inadequate number of toilets (*16*,*17*). Hospital staff and family caregivers can acquire infections through direct patient contact or contaminated fomites (*18*,*19*). Healthcare workers (i.e., doctors and nurses) have direct contact with patients; other staff, such as hospital cleaners, and visitors, who are not involved in patient care, might have contact only with hospital surfaces. Possible contamination of nearby hospital surfaces by Nipah patients with infectious bodily secretions, coupled with a lack of infection control measures in low-income hospitals, puts healthcare workers, caregivers, visitors, and other patients in the ward at risk for NiV infection by contaminated hospital surfaces. Propagation of a highly fatal pathogen with the capacity for person-to-person transmission within resource-constrained healthcare settings increases the risk for broader outbreaks (*11*,*20*).

In Bangladesh, resources for infection control in hospitals are severely limited (*16*), and we have limited knowledge about where to focus infection control to optimize use of scarce resources. Identification of fomites for possible NiV transmission would help design interventions prioritizing the area of hospital wards for disinfection to reduce surface contamination and possible risk for fomite transmission. Our objective was to identify whether Nipah patients contaminate nearby hospital surfaces with NiV RNA and, if so, which hospital surfaces are most commonly contaminated and which patients are most likely to contaminate their environment.

## Methods

### Case Identification and Sample Collection

We conducted this study in 3 Nipah surveillance hospitals at Faridpur, Rajshahi, and Rangpur, Bangladesh, during December 2013–April 2014. Surveillance physicians identified patients admitted with encephalitis, defined as fever or history of fever with axillary temperature >38.5°C (101.3°F) and altered mental status, new onset of seizures, or new neurologic deficit (*21*), and collected blood and oral swab samples. Because of resource constraints, surface sampling for all encephalitis cases was not possible; therefore, a research assistant swabbed hospital surfaces near encephalitis patients with a history of consuming raw date palm sap, contact with another encephalitis patient, or both (*22*). Occasionally, physicians from other nearby hospitals reported suspected Nipah case-patients to public health authorities. These patients also had biological samples collected for laboratory testing but were not included in the surface sampling study.

Blood samples were centrifuged at the local government health facility, and the separated serum was stored and transported to the Institute of Epidemiology Disease Control and Research laboratory in a liquid nitrogen dry shipper (−150°C) and then stored at −20°C until testing. From each patient, 1 oral swab was collected in 1 mL of nucleic acid extraction lysis buffer every consecutive day for 7 days, until hospital discharge or death, whichever occurred first.

A research assistant collected 1 swab sample from up to 5 areas near each patient: the wall beside patient bed, bed rail, bed sheet, clinical record file, and multipurpose towel used by family caregivers for cleaning patient secretions, drying hands, and other caregiving purposes. The research assistant collected surface swab samples at least 12 hours after the patient was admitted to the hospital. With 1 sterile rayon swab stick per surface, the research assistant swabbed the area of the wall in contact with the bed 45 cm high from the level of the bed sheet; all surfaces of the bed rail in the area near the patients’ head; half of the bed sheet where the patient’s head was, including underneath the patient; front and back covers of the patient file; and both sides of the multipurpose towel. Not all patients had a wall or bed rail near them because some patients were cared for on the floor and some were away from the walls. One swab sample per surface area was collected in separate cryovials with 1 mL of nucleic acid extraction lysis buffer (bioMérieux, Marcy-l’Étoile, France). The vials were kept in a cool box maintaining a temperature of 2°–8°C for up to 30 min after collection and then were placed in a liquid nitrogen dry shipper for storage and transportation.

### Testing of Clinical Samples and Surface Swab Samples

Serum samples were tested for NiV IgM using an IgM-capture enzyme immunoassay (*23*). Oral and surface swab samples were tested for NiV RNA by real-time reverse transcription PCR (rRT-PCR). Viral RNA was extracted using a Kingfisher Flex 96 (Thermo Scientific, Waltham, MA, USA) automatic extractor using InviMagVirus DNA/RNA Mini Kit/KF 96 (STRATEC Molecular, Birkenfeld, Germany). The rRT-PCR was performed on the CFX96 system (Bio-Rad, Inc., Hercules, CA, USA) and ABI7500 platform (Applied Biosystems, Foster City, CA, USA) using AgPath-ID One-Step RT-PCR Kit (Applied Biosystems). The following primers were used for detecting the NiV N gene: forward primer NVBNF2B 5′-CTGGTCTCTGCAGTTATCACCATC GA-3′, reverse primer NVBN593R 5′−ACGTACTTAGCC CAT CTT CTA GTTTCA-3′, and probe NVBN54P2 5′−Fam-CAG CTC CCGACACTGCCGAGG AT-BHQ–3′ (*24*). To provide evidence that similar viruses were present in human specimens and the environmental swab samples, we performed PCR-based direct sequencing using nucleic acids obtained from patients’ oral swab samples and their corresponding surface swab samples by using NiV-specific primers (Technical Appendix Table 1). The sequencing was performed using the ABI Big Dye Terminator v3.1 Cycle Sequencing Kit in an automated ABI 3500 XL genetic analyzer (both from Applied Biosystems). Nucleotide sequence similarity searches were performed using BLAST (https://www.ncbi.nlm.nih.gov/BLAST/).

**Table 1:** Laboratory results of swab samples of 6 patients with detectable Nipah virus RNA from 3 surveillance hospitals, Bangladesh, December 2013–April 2014*

Patient	Days after hospitalization collected and result														
					Surface swab sample										
					1						2				
	Oral swab sample				Towel	Bed sheet	Bed rail	Clinical file	Walls		Towel	Bed sheet	Bed rail	Clinical file	Walls
	1	2	3												
1	Pos	Pos									Pos	Neg	Pos	Neg	
2	Pos	Pos									Pos	Pos		Neg	Neg
3	Pos	Pos	Pos		Pos	Pos		Neg	Neg						
4	Pos					Pos	Neg	Neg							
5	Pos	Pos									Pos	Neg	Neg	Neg	Neg
6	Pos	Pos			Neg	Neg	Neg	Neg	Neg						

*Pos, positive; neg, negative. Blank cells indicate no sample collected.

### Community Investigation

An investigation team visited the communities of encephalitis patients identified at surveillance hospitals who had NiV IgM in serum to identify any other associated encephalitis cases. The team interviewed identified encephalitis patients and their caregivers using a structured questionnaire. Identified patients were asked about the nature of their contact with hospitalized patients (i.e., touching, being in the same room, feeding, sharing a bed, or cleaning body secretions) to find evidence of person-to-person transmission. The team also collected blood from the encephalitis patients identified in the community investigation.

### Classification of Cases

We classified an encephalitis case as laboratory-confirmed Nipah in a patient with NiV IgM in serum and a probable Nipah case as a case with an epidemiologic link with a laboratory-confirmed Nipah case in a person who died before blood could be collected for testing. We defined a Nipah spreader as a person with a probable or confirmed case who had close contact with at least 1 person in whom Nipah illness developed 5–15 days after contact (*5*).

### Statistical Analysis

We summarized the data using frequency and percentages. We assessed the difference in proportions using χ^2^ test or Fisher exact test when appropriate. We considered p<0.05 as statistically significant.

### Ethical Consideration

Study participants or their legal guardian provided informed written consent. The Ethical Review Committee of icddr,b (Dhaka, Bangladesh) reviewed and approved the study protocol. The Institutional Review Board at the Centers for Disease Control and Prevention (Atlanta, GA, USA) deferred to icddr,b’s approval.

## Results

**Figure 1:**
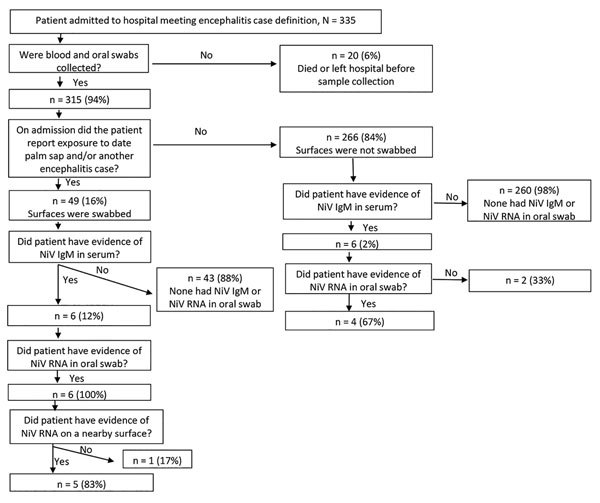
Number of blood samples, oral swab samples, and surface swab samples collected and tested from encephalitis patients identified in hospitals, Bangladesh, December 2013–April 2014.

Surveillance physicians identified 332 encephalitis cases in the 3 surveillance hospitals. One encephalitis case was reported from a nearby hospital, and we identified an additional 2 encephalitis cases from the community investigations. Of the 332 encephalitis cases identified in surveillance hospitals, we tested blood samples and oral swab samples from 312 (94%) case-patients and collected hospital surface swab samples from 49 case-patients who had a history of consuming raw date palm sap or contact with other encephalitis patients as reported by their caregiver on admission at the hospital. Of the 312 patients tested from surveillance hospitals, 9 (3%) had NiV IgM. All 3 case-patients identified during community investigations were hospitalized at nonsurveillance hospitals, and all had detectable NiV IgM (Figure 1). Through the community investigation, we identified an additional 4 probable Nipah case-patients who died before specimens could be collected. Thus, we identified a total of 16 Nipah cases from hospital and community investigations. Four cases occurred in isolation, but 12 clustered in 4 outbreaks. The 4 clusters comprised 8 laboratory-confirmed and 4 probable cases. Two of the 4 clusters involved person-to-person transmission (Technical Appendix).

**Figure 2:**
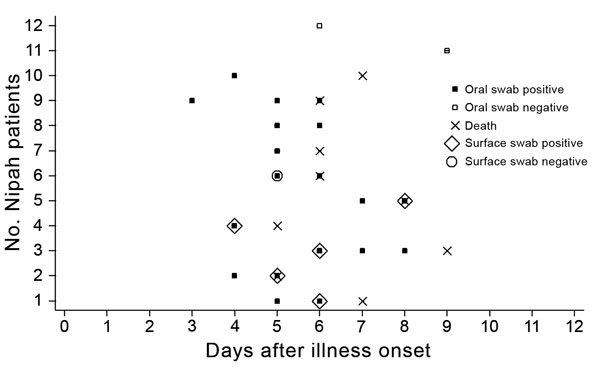
Timing of Nipah virus detection in oral swab and surface swab samples in relation to illness onset for 12 patients with laboratory-confirmed Nipah identified in hospitals, Bangladesh, December 2013–April 2014. Nearby surface swabs were not collected for 6 patients (nos. 7–12).

Of the 12 case-patients with laboratory-confirmed Nipah, 10 (83%) had NiV RNA in >1 oral swab sample (Figure 2). We collected 19 oral swab samples from these 10 case-patients; all 19 samples had evidence of NiV RNA. None of the 303 patients identified at surveillance hospitals without NiV IgM in serum had detectable NiV RNA on an oral swab sample. Of the 49 patients identified in surveillance hospitals for whom surface swab samples were collected, 6 had laboratory-confirmed Nipah (Table 1). We did not collect nearby surface swab samples from the other laboratory-confirmed Nipah patients with detectable NiV RNA in oral swab samples because during hospital admission they reported no history of consuming raw date palm sap or contact with other encephalitis patients. All of the 6 laboratory-confirmed Nipah patients from whom we collected nearby surface swab samples had detectable NiV RNA in their oral swab samples, and 5 of these had evidence of NiV RNA on >1 nearby surface. Of the 5 patients who contaminated nearby hospital surfaces, 4 contaminated their towels, 3 contaminated their bed sheets, and 1 contaminated the bed rail. We detected no evidence for NiV RNA-contaminated walls or clinical files (Table 2). 

**Table 2:** Proportion of surfaces contaminated with Nipah virus RNA associated with 6 laboratory-confirmed Nipah cases in 3 surveillance hospitals, Bangladesh, December 2013–April 2014*

Surface type	No. surface samples collected	No. (%) positive
Walls beside patient bed	4	0
Bed rails	4	1 (25)
Bed sheet	6	3 (50)
Clinical record file	6	0
Multipurpose towel	5	4 (80)

*Two patients were on the floor and had no bed rail surface; 2 patients did not have an adjacent wall; 1 patient did not have a towel sample.

We retrieved data on the partial N gene sequence (361 bp) from 4 patients’ oral swab samples and 3 surface swab samples surrounding 2 of these 4 patients: from the towel surface for 1 patient and the towel and bed rail for 1 patient (GenBank accession nos. KY887670–1, MF133373–6, and MF13337). The sequence recovery was 40% (4/10) for oral swab samples and 38% (3/8) for surface swab samples. The sequences from patients’ oral swab samples and corresponding surface swab samples were indistinguishable over the length of the sequenced fragments, and BLAST analysis indicated they were >99% similar to that of the NiV sequences (Gen­Bank accession nos. JN808857, JN808859, JN808860, JN808864, JN808862) reported from Bangladesh.

Our investigation identified 3 Nipah patients who were infected through person-to-person transmission. Two of these patients were infected by 1 probable case-patient who died before specimens were collected. The third case-patient had close contact with 2 laboratory-confirmed case-patients over the same time period, but we were unable to determine the source of infection. Both possible sources had evidence of NiV RNA in oral swabs; however, only 1 of the possible infectors contaminated the hospital surfaces and therefore might be more likely to be the infector (Technical Appendix Table 2, Figure 1). Laboratory-confirmed Nipah patients with detectable NiV RNA in oral swab samples were more likely to die than were patients with undetectable NiV RNA (90% [9/10] vs. 0% [0/2]; p = 0.04).

## Discussion

Nipah patients frequently contaminated hospital surfaces near them with detectable NiV RNA, posing a risk for fomiteborne Nipah transmission. The most commonly contaminated surfaces were the bed sheets and the towels used by caregivers for patient care. In Bangladesh, family caregivers, rather than trained healthcare workers, provide 24-hour hands-on care to hospitalized patients (*17*,*25*). The more severe the patient’s illness, the more hands-on care he or she receives (*17*). Most Nipah patients in Bangladesh are unconscious when they are brought in for care and have cough and difficulty breathing (*21*), requiring close attention and care. Nipah patients often dribble frothy oral secretions, soiling themselves and contaminating their bed sheets. Caregivers frequently use a towel brought from home to clean patient oral secretions (*17*) and often use the same towel throughout the hospital stay. They also frequently use the same towel for cleaning their own hands and face. The lack of running water in healthcare settings in Bangladesh makes it difficult for caregivers to wash their hands or wash the items used for patient care (*16*). One Nipah patient we identified was infected after caring for 2 other patients, 1 of whom had a towel contaminated with detectable NiV RNA, highlighting the possibility of this fomite as a vehicle of NiV transmission from patient to caregiver. The caregiver also contaminated nearby surfaces during his illness, including the towel, although no further transmission was evident.

We did not detect NiV RNA on the patient clinical file and nearby wall surfaces, most likely because of the distance and infrequency of contact of these surfaces with a patient’s oral secretions. Although in Bangladesh hospitals patient clinical files are commonly kept on the bed or under the bed sheet or pillow, they are also sometimes kept at the nurses’ station, reducing the frequency of the file coming into contact with patient oral secretions. We also found that the walls were the surfaces farthest from the patient and for this reason might have been less frequently contaminated with oral secretions.

Transmission of NiV through fomites is plausible. Many paramyxoviruses, including respiratory syncytial virus, parainfluenza viruses 1–4, and human metapneumoviruses, have been identified on hospital surfaces, and fomiteborne transmission of these pathogens has been reported (*26*–*30*). Past studies have indicated that other paramyxoviruses can survive on surfaces for up to 10 hours and be a source of infection for patients, healthcare workers, and hospital visitors (*26*,*31*–*33*). Animal experiments with NiV in a hamster model also showed that NiV can be transmitted through fomites (*34*). Although it is not known how long NiV remains infectious in the environment, we hypothesize that surfaces might play an important role in NiV transmission for several reasons: hospital surfaces in Bangladesh are not routinely cleaned (*16*); new patients frequently use the same bed sheets used by previous occupants (*16*); caregivers and healthcare workers frequently come into contact with contaminated surfaces (*16*); and handwashing by caregivers and healthcare workers occurs infrequently because of several barriers, including a lack of running water in hospitals (*16*,*17*).

Investigations of earlier outbreaks showed that only 7% of all Nipah patients were Nipah spreaders (*5*,*35*). During our 5-month study, we identified 16 Nipah patients and 2 likely spreaders. The 2 spreaders we identified both infected their primary caregivers (Technical Appendix Table 2, Figure 1). This finding provides additional evidence that exposure to contaminated oral secretions drives person-to-person transmission of NiV. Caregivers can be exposed to oral secretions through direct patient contact, contaminated surfaces, or both. Family care providers maintained close physical contact with Nipah patients, including sharing eating utensils and drinking glasses, sleeping in the same bed, and hugging and kissing near the time of death, which highlights that contact transmission might play a major role in NiV transmission (*36*). Our investigation showed similar patterns of caregiving practices in this outbreak (Technical Appendix Table 2). Reports from earlier outbreaks also demonstrated that Nipah patients who had respiratory involvement (difficulty breathing and cough) were more likely to become Nipah spreaders (*5*,*6*,*9*,*12*). However, our current understanding is limited about why some Nipah patients shed NiV in their oral secretions (and for how long they shed) but others do not. Virus replication in the respiratory epithelium of hamsters infected with a high dose of NiV was 2 logs higher than in those infected with a low dose, suggesting dose of exposure might affect viral shedding in respiratory secretions (*36*). All NiV case-patients who had evidence of NiV RNA in their oral secretions died, and those without NiV RNA survived, suggesting that virulence also might be associated with tissue tropism or viral load. A better understanding of the factors that determine variations of viral shedding between Nipah patients might explain the drivers of person-to-person transmission of NiV. Given limited resources for infection control in low-income settings, early identification of patients who shed NiV could help focus resources to reduce subsequent transmission of NiV from person to person. NiV surveillance in Bangladesh relies on a central laboratory located in the capital city; thus, confirming a diagnosis can take several days or weeks and limits the ability for an early intervention. A rapid diagnostic test that could quickly identify NiV patients at the bedside could be a powerful tool in the early identification of potential NiV spreaders, formulating early intervention and thereby preventing NiV transmission in hospitals.

Our study had limited power to detect a significant difference in characteristics of patients with and without detectable NiV RNA in oral swabs because of the small number of laboratory-confirmed Nipah patients we identified. However, despite low power and small number of observations, we found a significant association between having detectable NiV RNA in an oral swab sample and dying from illness. In addition, although we identified NiV RNA on various surfaces, the presence of nucleic acid does not confirm contamination with a viable virus nor does it indicate that fomites are important for NiV transmission. However, laboratory evidence suggests that paramyxoviruses can survive on surfaces and have been a source of transmission in healthcare settings (*26*,*27*). Studies on NiV survival in environmental condition have shown that NiV survival varies from a few hours to ≈2 days and is highly dependent on pH, temperature, and desiccation (*37*,*38*). Previous studies suggest that persons at highest risk for infection from patients with NiV are family caregivers who provide continuous care, even during hospitalizations (*9*,*10*,*20*). Therefore, even if the virus remains viable for only a short time, it still could pose a major risk for these caregivers.

Efforts to reduce the risk for person-to-person NiV transmission in healthcare settings should target patient caregiving practices related to the use of towels. Resources are limited for hospitals and for patients’ families; however, affordable options exist that deserve additional investigation to determine their acceptability and feasibility. For example, families could be counseled to purchase a separate towel for patients, which costs ≈US $0.50. Also, hospitals could provide low-cost disinfectants, such as 0.5% sodium hypochlorite, for ≈US $1/liter to use to disinfect towels and caregiver hands. We also advocate for the development of a rapid test to identify NiV patients, who represent only ≈3% of all encephalitis patients, to most efficiently focus infection control efforts for NiV prevention (*6*,*39*,*40*).

Technical Appendix Nipah virus–specific primers, epidemiologic link between cases within each Nipah virus cluster, and clustered and isolated case-patients, Bangladesh, December 2013–April 2014.
