# Supplementary material for: Nipah Virus Contamination of Hospital Surfaces during Outbreaks, Bangladesh, 2013–2014
Source: Emerg Infect Dis. 2018 Jan;24(1):15–21. doi: 10.3201/eid2401.161758 (PMC5749460; doi:10.3201/eid2401.161758)
Supplement: Technical Appendix — Nipah virus–specific primers, epidemiologic link between cases within each Nipah virus cluster, and clustered and isolated case-patients, Bangladesh, December 2013–April 2014. [file 16-1758-Techapp-s1.pdf]

Article DOI: https://doi.org/10.3201/eid2401.161758

Nipah Virus Contamination of Hospital Surfaces during Outbreaks, Bangladesh, 2013–2014

Technical Appendix

Technical Appendix Table 1. NiV-specific primers and rRT-PCR reaction conditions for partial NiV-N gene sequencing*

Primer Name	Sequence (5'-3')	Location (Amplicon Size)	Reaction Condition
NiV-N-299F<br>NiV-N-661R	AGTGCTGCCGAGTCAATGAA<br>TCGGGAGCTGTAACTGCTTT	299-661 (361bp)	95 C for 10 min, 40 cycles (95 C for 30 sec, 50 C for 30 sec, 72 C for 45 sec) 72 C for 10 min

*All nucleotide positions based on reference sequence NC_002728 from GenBank. NiV, Nipah virus.

Technical Appendix Table 2. Epidemiologic link between cases within each NiV cluster, Bangladesh, December 2013–April 2014

Cluster	Patients	Relationship among patients	Nature of contact	Drank raw/fermented date palm sap	Survived
A	1, 11, 12, 13†	1 and 13: neighbors<br>11: wife of 13<br>12: daughter of 11 and 13	11 and 12 became ill after their contact with 13. 11 was involved in feeding, cleaning body secretions, and comforting her husband (13) during his illness at home and hospital. While at home, she also shared a bed with him during his illness. During 13's illness, his 10-mo-old daughter (12) spent most of her time with him, stayed in the same room, slept in the same bed, and reportedly hugged and kissed him frequently 11 also cared for her daughter, provided nursing and feeding, and became ill 3 d after her daughter became ill.	1, 13 from same source	11, 12
B	3, 6, 5, 14†	3 and 5: cousins<br>6: nephew of 3, and 5<br>14 and 6: neighbors	5 became ill after contact with 3 and 6. 5 helped 3 and 6 during transportation to the hospital and during the hospital stay and became ill 2 wks after 3 and 6 had illness onset. 3 and 6, reportedly had no contact with each other or any other encephalitis patients, became ill 1 day apart. 14 denied any contact with other patients in the cluster or any other encephalitis patients.	14, 3, and 6 from same source	None
C	4, 15†	Neighbors	No contact with each other after illness onset.	Both from same source	None
D	10, 16†	Neighbors	No contact with each other after illness onset.	Both from same source	None
-	2, 7, 8, 9	Isolated	No contact	2,7,8 each from different source,9 had no reported history of DPS consumption	8

*NiV, Nipah virus.
†Probable case-patient.

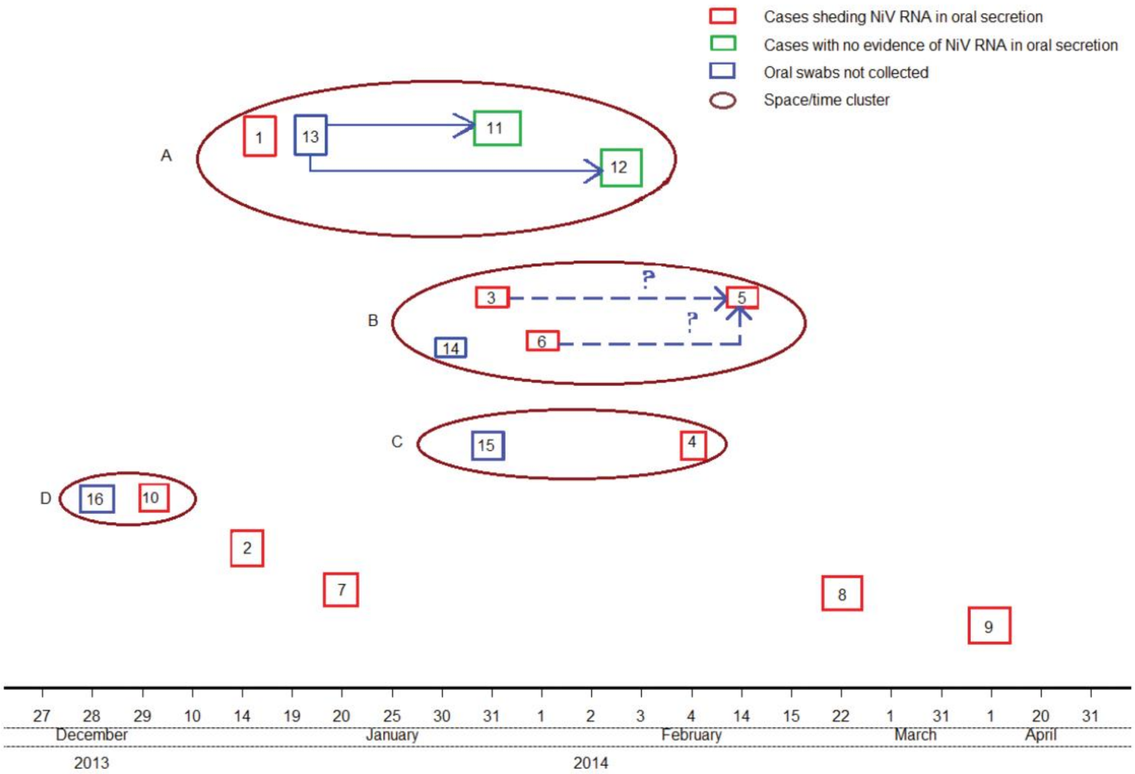

Technical Appendix Figure. Clustered and isolated Nipah patients with dates of illness onset and NiV RNA status in oral swab samples, Bangladesh, December 2013–April 2014. Each letter indicates a cluster and each number an individual Nipah patient. Case 13–16 were probable cases; all others were confirmed cases. Illness for patients 1, 3–6 and 11–16 occurred in clusters; 2 and 7–9 were isolated. Note: The solid line between cases indicates person-to-person transmission, the dotted line indicates unknown infector. The time axis was not scaled.
